# Supplementary material for: Multi-omics investigation of Porphyromonas gingivalis exacerbating acute kidney injury through the gut-kidney axis
Source: mSystems. 2025 Jan 14;10(2):e01136-24. doi: 10.1128/msystems.01136-24 (PMC11834432; doi:10.1128/msystems.01136-24)
Supplement: Supplemental material — Fig. S1-S7, Table S1, and supplemental methods and materials. [file msystems.01136-24-s0001.docx]

**Multi-Omics Investigation of *Porphyromonas gingivalis* Exacerbating Acute Kidney Injury through the Gut-Kidney Axis**

**Methods and Materials**

**Renal Function**

Serum was obtained from whole blood samples for renal function testing. The assays for urea, creatinine, and uric acid levels were conducted using specific kits from Servicebio, Wuhan, China.

**Liquid chromatography-mass spectrometry (LC-MS) metabolomics analysis**

Serum samples were analyzed for untargeted metabolomics by Shanghai Personal Biotechnology Co., Ltd. After thawing at 4°C and vortexing to mix, the samples were treated with methanol, centrifuged, and the supernatant was collected. The dried extract was reconstituted in a solution for LC-MS analysis. The analysis was performed using a Vanquish UHPLC System (Thermo Fisher Scientific) and metabolites were detected with an Orbitrap Exploris 120 (Thermo Fisher Scientific) using ESI ionization. Data were processed with ProteoWizard software, annotated through a mass spectrometry database, and analyzed using R software for principal component analysis (PCA) and differential metabolite identification. Significant metabolites were determined using P values and VIP scores and visualized with heatmaps and volcano plots. Pathway enrichment and topology analyses were also conducted.

**Transcriptome sequencing**

Transcriptome sequencing was performed by Guangdong Magigene Biotechnology Co., Ltd. Total RNA was extracted from renal tissues, and quality control assays were conducted. First-strand cDNA was synthesized using these mRNA fragments as templates with random hexamer primers, followed by second-strand cDNA synthesis. PCR amplification was performed, and the final sequencing library was purified and quality assessed. Sequencing was carried out on the Illumina HiSeq 2500 platform using the PE150 strategy. Raw image data were converted to sequencing reads and stored in FASTQ format, which includes sequence and quality information.

**Measurement of Indole-3-acetic acid (3-IAA)**

For the quantification of the serum concentration of 3-IAA, the mouse serum was diluted with PBS, and the chemiluminescence immune assay (Abbexa, Cat#abx190011) was carried out in accordance with the manufacturer's protocol.


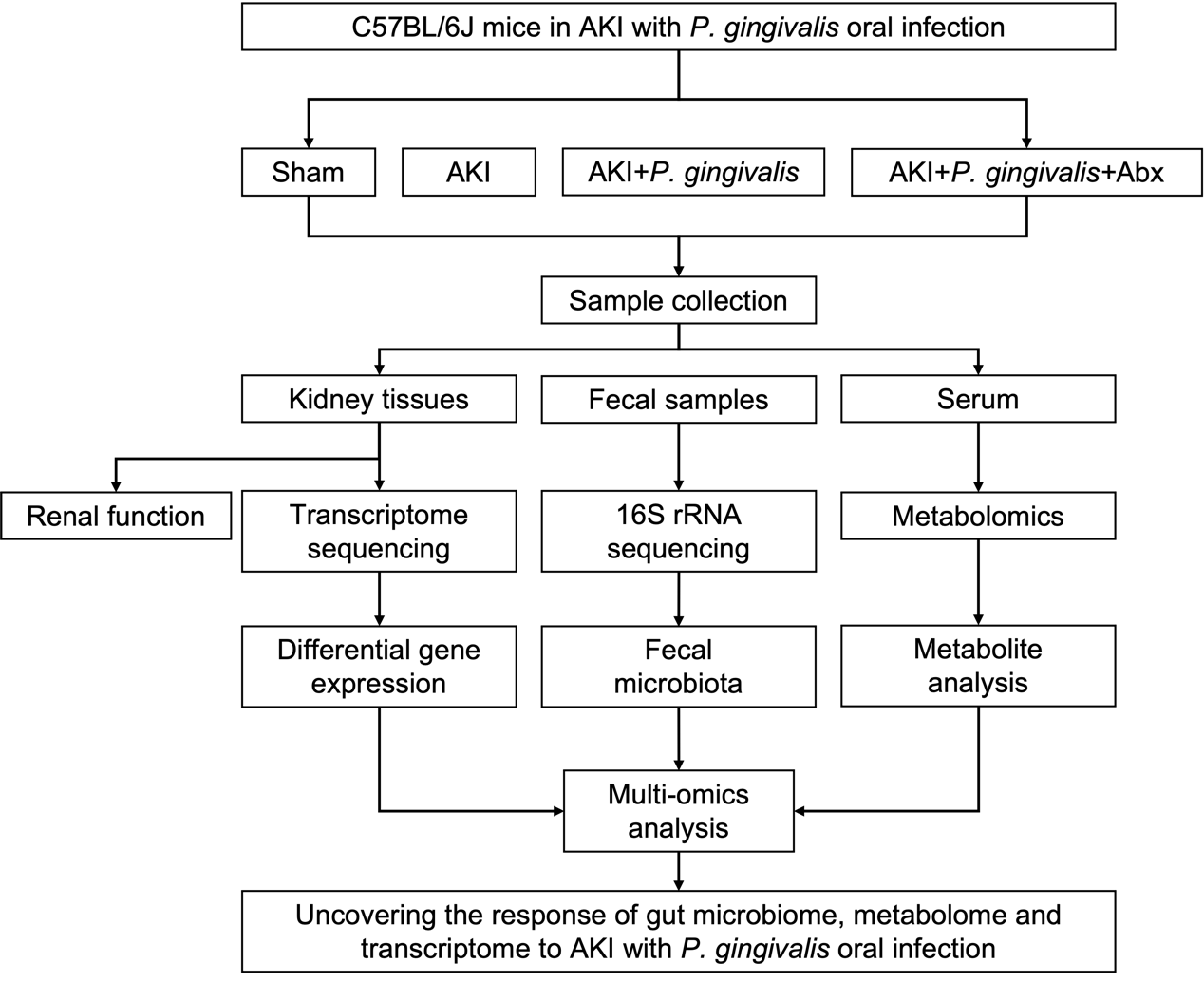


**Supplemental Figure 1. Experimental flowchart.**

**
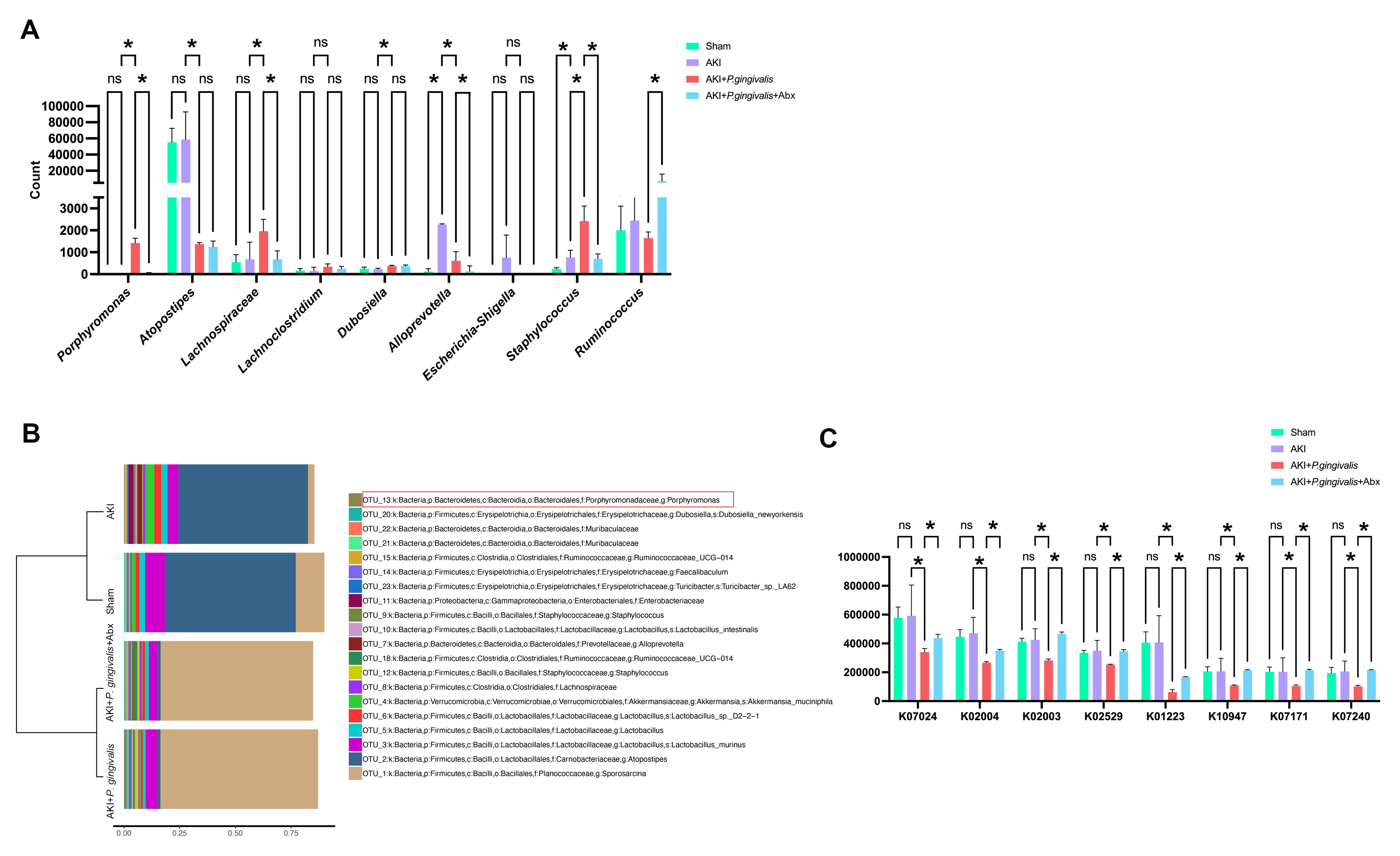
Supplemental Figure 2. Analysis of gut microbiota in mice. (A)** Comparisons of the major gut microbiota at the genus level. Statistical significance was determined using a one-way ANOVA test with Scheffé's post-hoc analysis. ns, not significant; *, P < 0.05; **, P < 0.01; ***, P < 0.001; ****, P < 0.0001. **(B)** The left clustering tree represents a phylogenetic analysis, while the right panel exhibits the relative abundance distribution of species at the OTU level for each group. **(C)** Functional prediction analysis based on 16S rRNA sequencing data was performed. Statistical significance was assessed using a one-way ANOVA test with Scheffé's post-hoc analysis: ns, not significant; *, P < 0.05; **, P < 0.01; ***, P < 0.001; ****, P < 0.0001.

**
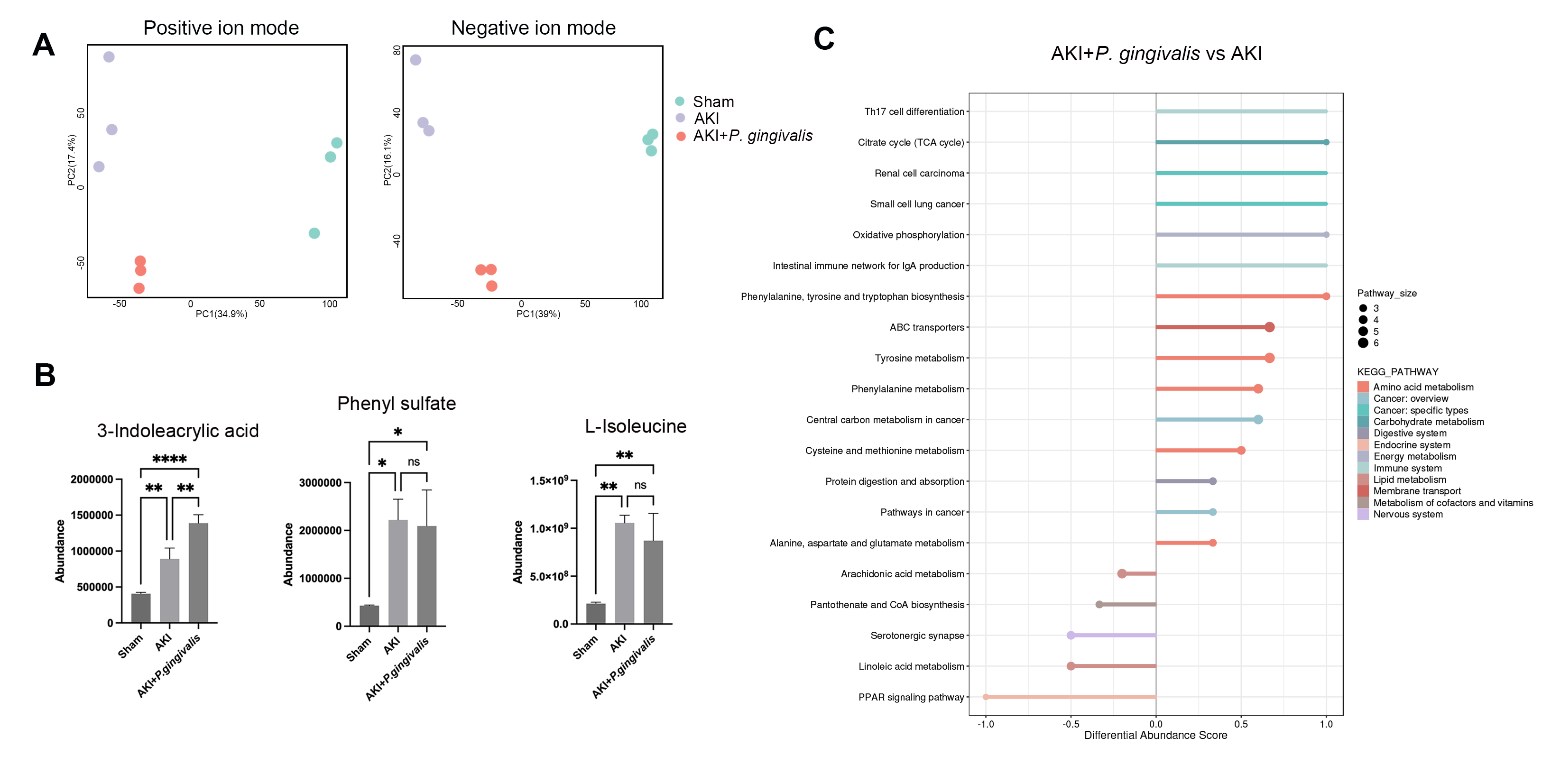
Supplemental Figure 3. Analysis of serum metabolites in mice. (A)** Principal component analysis (PCA) of serum metabolites in mice, with the left panel depicting the positive ion mode and the right panel showing the negative ion mode. **(B)** Comparisons of serum metabolites. Statistical significance was evaluated using a one-way ANOVA test with Scheffé's post-hoc analysis: ns, not significant; *, P < 0.05; **, P < 0.01; ***, P < 0.001; ****, P < 0.0001. **(C)** Kyoto Encyclopedia of Genes and Genomes (KEGG) pathway enrichment analysis was conducted on the list of differential metabolites in the serum of the AKI+*P. gingivalis* group compared to the AKI group. The x-axis represents the DA-score value, calculated as (number of upregulated metabolites - number of downregulated metabolites) / total number of differential metabolites in the pathway. The y-axis lists the metabolic pathways.

**
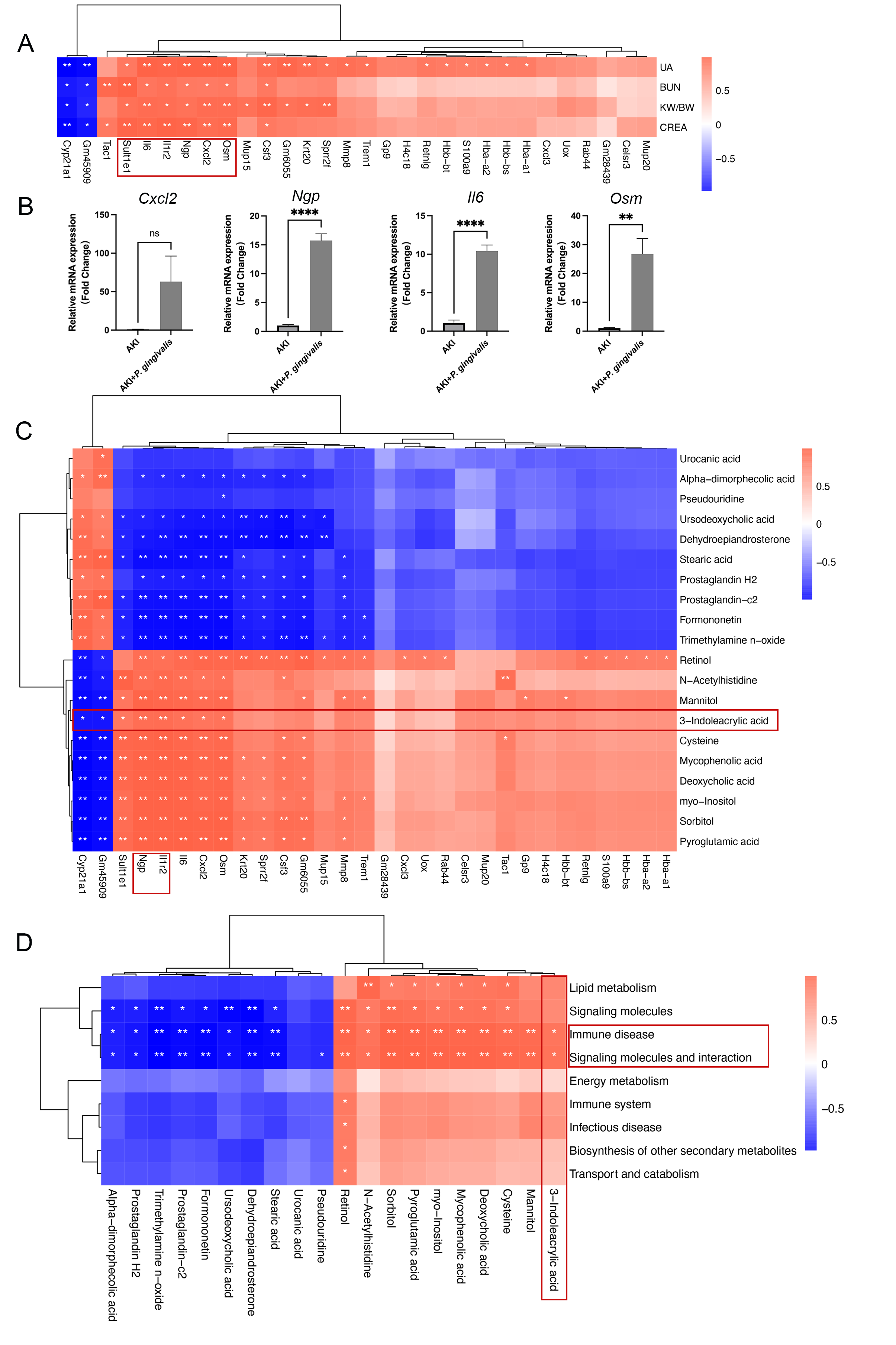
****Supplemental Figure 4. Analysis of serum metabolites and renal transcriptome in mice. (A)** Correlation analysis was conducted to assess the relationships between renal function parameters and top 30 differentially expressed renal tissue genes in the AKI+*P. gingivalis* group compared to the AKI group. **(B)** qRT-PCR analysis was performed to assess the expression levels of *Cxcl2*, *Ngp*, *Il6*, and *Osm* gene expression in renal tissue, normalized to *Gapdh*. **(C)** Correlation analysis was performed to explore the associations between top 20 differential serum metabolites and top 30 differentially expressed renal tissue genes in the AKI+*P. gingivalis* group compared to the AKI group. **(D)** After conducting a KEGG pathway enrichment analysis on the differentially expressed renal tissue genes, a correlation analysis was further performed to investigate the relationships between the top 20 differential serum metabolites and the primary KEGG pathways enriched.


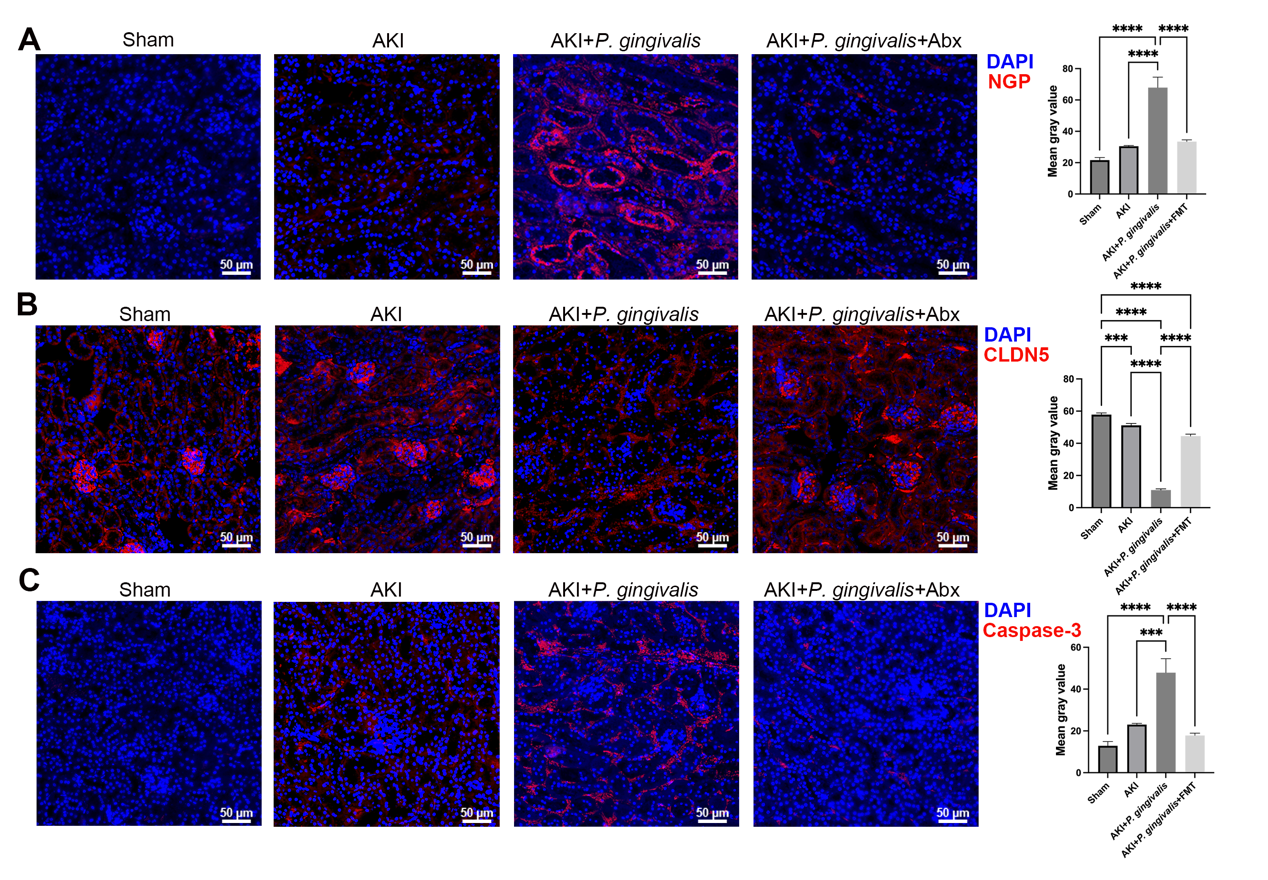


**Supplemental Figure 5.** **(A-C)** Immunofluorescence staining for NPG, CLDN5, and Caspase-3 in renal tissue and quantitative analysis. The results are expressed as the mean ± SD. *, P < 0.05; **, P < 0.01; ***, P < 0.001; ****, P < 0.0001 by ANOVA.


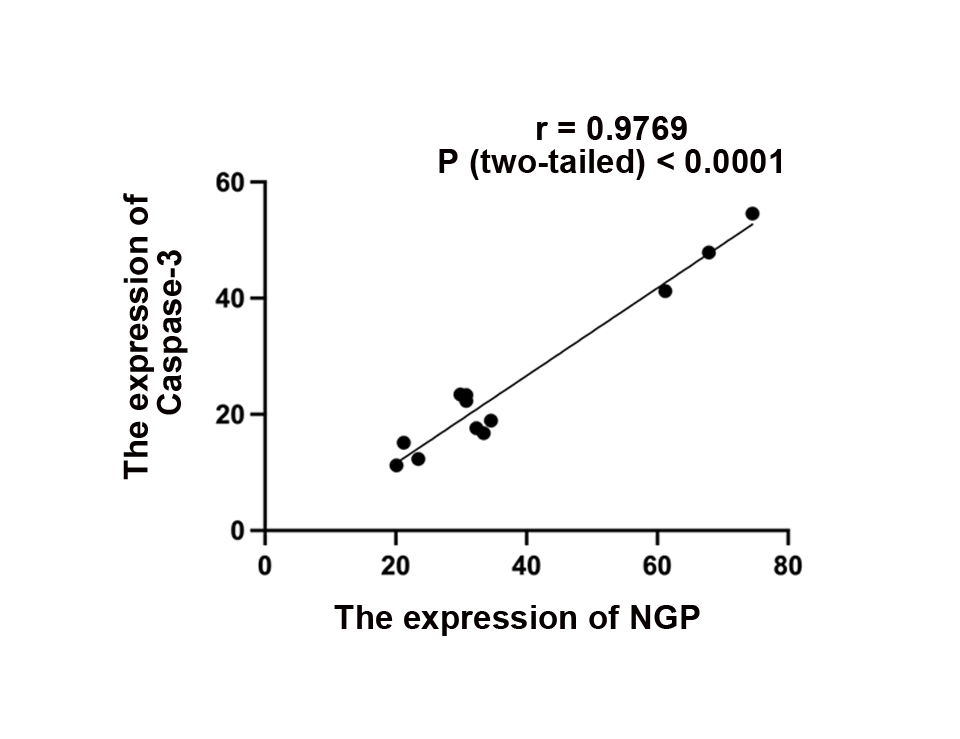


**Supplemental Figure 6. The elevation of NGP expression was accompanied by the augmentation of caspase - 3 expression in kidney tissue.** A correlation analysis was conducted to evaluate the relationship between the mean gray value of NPG fluorescence staining and that of caspase - 3 fluorescence staining in the kidney tissue of mice in each group.


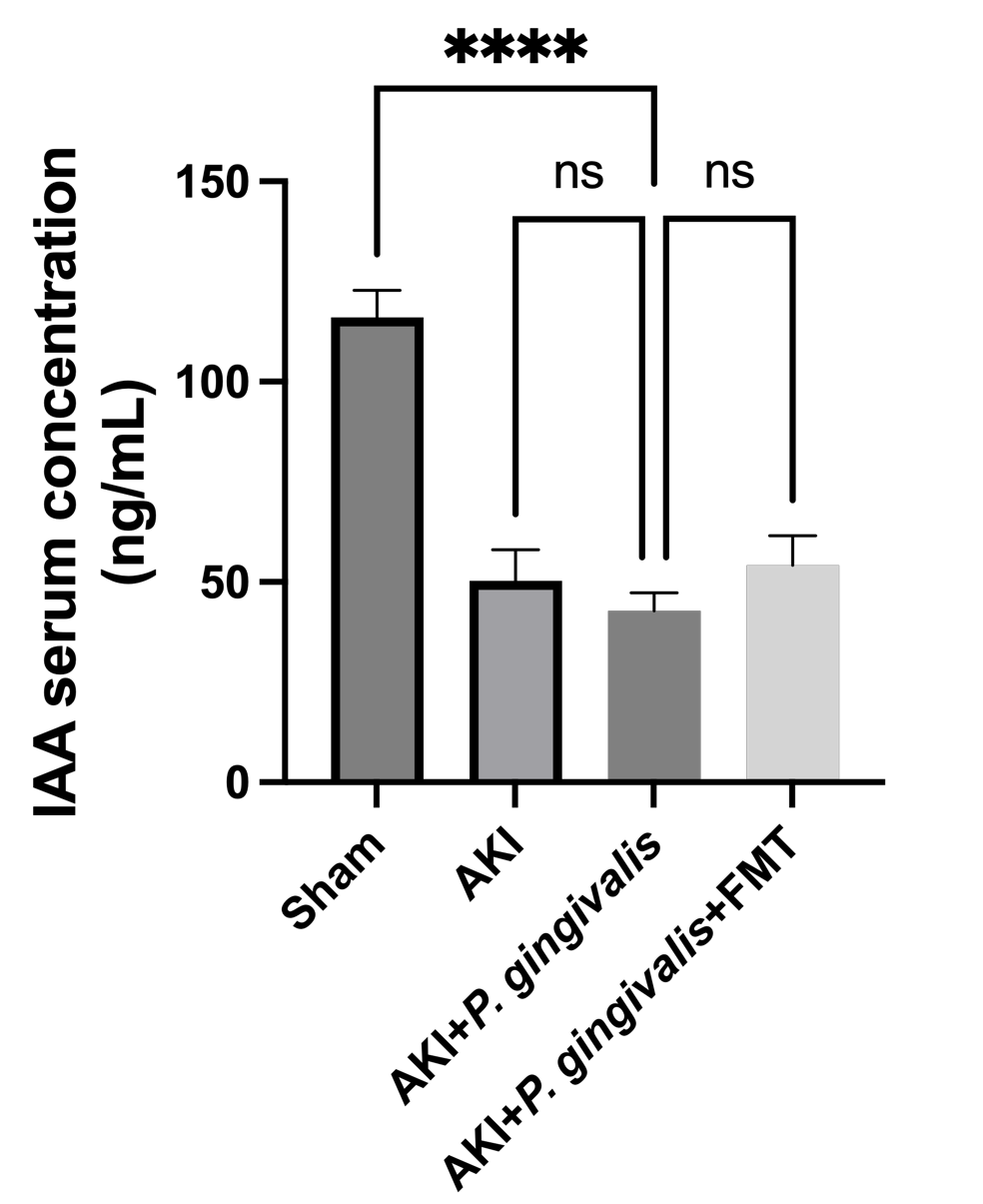


**Supplement Figure 7. The concentrations of 3-IAA in the serum of mice in each group.** The results are expressed as the mean ± SD. *, P < 0.05; **, P < 0.01; ***, P < 0.001; ****, P < 0.0001 by ANOVA.

**Supplemental Table 1 Summary characteristics**

| Parameter | Group | mean ± SEM | ANOVA  P value | F | R  squared | Significance |
| --- | --- | --- | --- | --- | --- | --- |
| KW/BW (%) | Sham | 1.806 ± 0.0525 | ＜0.001 | 37.45 | 0.8753 |  |
|  | AKI | 2.484 ± 0.1137 |  |  |  | a |
|  | AKI+*P. gingivalis* | 3.528 ± 0.1072 |  |  |  | a, b |
|  | AKI+*P. gingivalis*+Abx | 2.644 ± 0.1626 |  |  |  | a, c |
| BUN (mg/dL) | Sham | 10.73 ± 0.4525 | ＜0.001 | 111.7 | 0.9599 |  |
|  | AKI | 41.14 ± 1.2786 |  |  |  | a |
|  | AKI+*P. gingivalis* | 56.82 ± 2.1202 |  |  |  | a, b |
|  | AKI+*P. gingivalis*+Abx | 43.92 ± 1.5019 |  |  |  | a, c |
| CREA (μmol/L) | Sham | 23.98 ± 2.2755 | ＜0.001 | 64.40 | 0.9324 |  |
|  | AKI | 44.07 ± 1.8526 |  |  |  | a |
|  | AKI+*P. gingivalis* | 77.21 ± 3.0933 |  |  |  | a, b |
|  | AKI+*P. gingivalis*+Abx | 49.28 ± 2.3222 |  |  |  | a, c |
| UA (μmol/L) | Sham | 49.10 ± 4.1729 | ＜0.001 | 55.53 | 0.9225 |  |
|  | AKI | 130.4 ± 3.6760 |  |  |  | a |
|  | AKI+*P. gingivalis* | 194.8 ± 5.0476 |  |  |  | a, b |
|  | AKI+*P. gingivalis*+Abx | 138.0 ± 7.9282 |  |  |  | a, c |

After performing ANOVA analysis, significant differences were observed among the various groups. Specifically, the "a" was used to represent statistically significant differences when each group was compared to the Sham group. The "b" indicated a significant difference specifically between Group AKI and Group AKI+*P. gingivalis*. The "c" signified a significant difference between Group AKI+*P. gingivalis* and Group AKI+*P. gingivalis*+Abx.
